# Correlates of HIV Prevention Advocacy by Persons Living with HIV in Kampala, Uganda: A Cross-sectional Evaluation of a Conceptual Model

**DOI:** 10.1007/s12529-024-10277-3

**Published:** 2024-03-22

**Authors:** Glenn J. Wagner, Laura M. Bogart, Joseph K. B. Matovu, Stephen Okoboi, Violet Gwokyalya, Susan Ninsiima, Ryan K. McBain, Erik D. Storholm, Nipher Malika, Harold D. Green

**Affiliations:** 1 RAND Corporation, 1776 Main Street, Santa Monica, CA 90407 USA; 2 Makerere University School of Public Health, Kampala, Uganda; 3 Infectious Diseases Institute, Makerere University College of Health Sciences, Kampala, Uganda; 4 San Diego State University, San Diego, CA USA; 5 University of Indiana Bloomington School of Public Health, Bloomington, IN USA

**Keywords:** HIV, Prevention, Advocacy, Disclosure, Self-efficacy

## Abstract

**Background:**

HIV prevention advocacy empowers persons living with HIV (PLWH) to act as advocates and encourage members of their social networks to engage in protective behaviors such as HIV testing, condom use, and antiretroviral therapy (ART) adherence. We examined correlates of HIV prevention advocacy among PLWH in Uganda.

**Method:**

A cross-sectional analysis was conducted with baseline data from 210 PLWH (70% female; mean age = 40 years) who enrolled in a trial of an HIV prevention advocacy training program in Kampala, Uganda. The baseline survey, which was completed prior to receipt of the intervention, included multiple measures of HIV prevention advocacy (general and specific to named social network members), as well as internalized HIV stigma, HIV disclosure, HIV knowledge, positive living (condom use; ART adherence), and self-efficacy for HIV prevention advocacy.

**Results:**

Consistent with our hypotheses, HIV disclosure, HIV knowledge, consistent condom use, and HIV prevention advocacy self-efficacy were all positively correlated with at least one measure of HIV prevention advocacy, after controlling for the other constructs in multiple regression analysis. Internalized HIV stigma was positively correlated with advocacy in bivariate analysis only.

**Conclusion:**

These findings identify which characteristics of PLWH are associated with acting as change agents for others in their social network to engage in HIV protective behaviors.

## Introduction

In Uganda, HIV prevalence has plateaued at about 6% among those aged 15–64 [1], necessitating the need for innovative solutions to promote HIV prevention. One promising approach is to empower persons living with HIV (PLWH), who are successfully managing their HIV disease, to act as change agents by encouraging members of their social networks to engage in HIV protective behaviors (e.g., HIV testing, condom use, adherence to HIV antiretroviral therapy). Interventions based on this kind of peer advocacy have been effective at increasing disease prevention behaviors in the context of HIV [2–5].

Research suggests that as PLWH receive antiretroviral therapy (ART) and stabilize their health, they are motivated to encourage peers, friends, and family to reduce HIV risk and to seek HIV testing and care [6, 7]. PLWH can be influential and credible in conveying prevention messages to their network members, given their close relations and their ability to exemplify the benefits of HIV testing and care on health [8]. With effective advocacy training, mobilizing PLWH to be change agents within their networks has the potential to make a significant contribution to HIV prevention, particularly in high-prevalence settings such as Uganda.

Building on the concepts of social diffusion [9], cognitive consistency [10], and social influence [11], we developed a conceptual framework for how PLWH can serve as change agents for others in their social network to engage in HIV protective behaviors. These concepts suggest that behavior change can be initiated by a few and diffused to others through modeling, advocacy, and shifts in social norms.

**Fig. 1:**
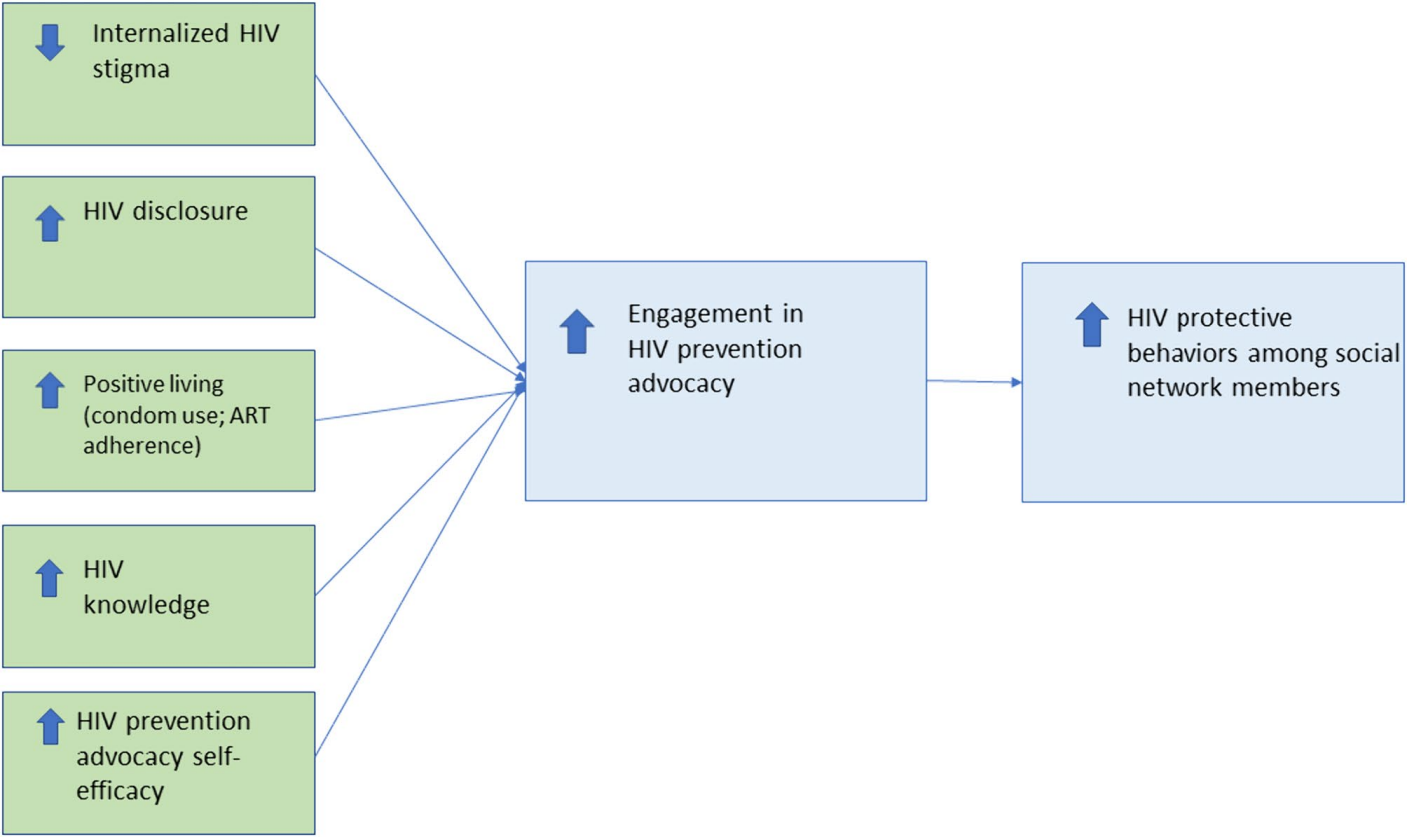
Conceptual framework for promotion of HIV prevention advocacy to affect HIV prevention advocacy to affect HIV protective behaviors among social network members

Our framework (see Fig. 1) posits that self-acceptance, through reduced internalized HIV stigma (i.e., the process by which people apply negative stereotypes about PLWH to themselves; [12]), helps facilitate comfort with disclosing one’s HIV status and sharing one’s experience of living with HIV [13]. This comfort with HIV disclosure and discussing HIV serves to promote engagement in HIV prevention advocacy [14]. To be comfortable and effective in advocating for HIV protective behaviors, PLWH must prioritize positive living and model the behaviors they encourage others to adopt (e.g., sexual risk reduction, ART adherence) [4, 5]. Furthermore, HIV knowledge is important for accurate advocacy and dispelling myths and misconceptions. Lastly, learning communication skills and strategies for when, how, and who to engage in advocacy helps to bolster confidence and self-efficacy for conducting advocacy [15].

The conceptual model described above highlights the components that are hypothesized to be important for building advocacy skills and self-efficacy. Self-efficacy regarding a particular behavior has been shown to be an important precursor to enacting that behavior [16]. Ultimately, HIV prevention advocacy by PLWH within their social networks is hypothesized to result in increased HIV protective behaviors in the network and larger community.

This conceptual framework forms the basis of a network-based advocacy group intervention, *Game Changers for HIV Prevention* (GC-HIV), that mobilizes PLWH to act as change agents for HIV prevention within their social networks [4]. The efficacy of the intervention for increasing HIV protective behaviors within social networks is currently being evaluated in an ongoing randomized controlled trial. We used baseline data from the trial to examine correlates of HIV prevention advocacy from among the constructs in our conceptual model, as well as correlates between the constructs within the model.

We hypothesized that engagement in prevention advocacy would be associated with lower internalized HIV stigma, and greater levels of HIV disclosure, HIV knowledge, positive living, and self-efficacy related to advocacy. Furthermore, we hypothesized that internalized HIV stigma would be negatively correlated with HIV disclosure and self-efficacy related to advocacy, and that HIV knowledge would be positively correlated with positive living and self-efficacy for advocacy.

## Methods

### Study Design

We conducted a cross-sectional analysis with baseline data from a randomized controlled trial of an intervention that empowers PLWH to engage in HIV prevention advocacy with members of their social network. The study protocol was approved by the Infectious Diseases Institute Research Ethics Committee and the Human Subjects Protection Committee at the RAND Corporation, and registered with the Uganda National Council of Science and Technology. Further details of the study protocol are available in a prior publication [4].

### Participants

Recruitment took place between January 2022 and February 2023 in Kampala, Uganda at The Infectious Diseases Institute (IDI), which provides outpatient HIV care to over 8000 PLWH. Eligibility criteria included (1) age ≥ 18 years; (2) living with HIV; (3) in HIV care for at least 1 year (because they are more likely to be medically stable, adjusted to their HIV diagnosis, and have disclosed to several people, and thus more likely to be ready to engage in advocacy); (4) did not participate in the pilot study of the intervention [17]; (5) speak fluent Luganda; (6) health status sufficiently stable (based on medical chart review) to complete the 18-month study; (7) no signs of significant cognitive impairment (based on interviewer observation); and (8) partner/spouse or household member living with HIV is not already enrolled in the study.

To recruit PLWH (referred to as “index” participants), the study coordinator gave a brief talk in the IDI waiting room at the start of each recruitment day, describing the nature of the study and participation involvement. Interested clients were asked to reveal themselves to the coordinator, who then conducted a formal screening of their eligibility. This was followed by the informed consent process (including obtaining written informed consent) and administration of the baseline assessment.

### Measures

The survey assessment was interviewer-administered using Network Canvas software [18] and conducted in Luganda. Measures had been translated from English to Luganda using standard translation/backtranslation methodology during a prior study [17]. All measures were developed by the study team, except those in which an attribution is cited below. Participants received between 30,000 and 70,000 Uganda Shillings (~$20 USD) after completing the assessment. This amount was for the purpose of covering the cost of transportation, and the amount varied based on the distance travelled.

In addition to survey measures, each participant was asked to list 20 people in their social network (“alters”) with whom they interact most frequently. For each alter, we gathered information to assess network composition, including knowledge of respondent’s HIV status, perceived HIV status, and use of HIV care and ART if believed to be living with HIV. Our prior research shows that alter health behaviors that are related to HIV can be accurately reported by index participants who are PLWH [19].

*HIV prevention advocacy* to assess *general HIV prevention advocacy* engagement, participants were asked to report how much they discussed each of 10 different areas of HIV prevention (e.g., HIV testing, condom use, fewer sex partners, engage in HIV care, use of pre-exposure prophylaxis (PrEP)) with people they know. The time frame for these discussions was the past 3 months, and response options ranged from 1 “not at all” to 5 “very much.” The mean score across all items was calculated. The scale displayed high internal reliability (Cronbach’s alpha = .89).

We also assessed five measures of *alter-specific advocacy* by asking participants about advocacy conducted specifically with the alters named in the social network assessment. For each alter named, the participant was asked if they had talked with the alter about condom use (all alters), HIV testing and PrEP (in separate items if the alter was not HIV-positive), and engagement in HIV care and ART adherence in separate items (if the alter was believed to be living with HIV), in the past 3 months. For each of these items, if discussions with the alter had taken place, follow-up questions were asked to assess whether advocacy had included the following actions to promote the alter’s use of the behavior: (1) encouraged the alter; (2) provided information (e.g., where and how to get access); and (3) provided direct support (e.g., accompanied alter to the clinic). The response option for each of these three questions was 0 “no” or 1 “yes.” The mean across the three items was calculated for each alter, followed by the mean across all alters named by the participant. The latter means constitute alter-specific measures of advocacy related to condom use, HIV testing, PrEP, HIV care, and ART adherence.

For the analysis, we examined five measures of HIV prevention advocacy: general HIV prevention advocacy and four alter-specific measures of advocacy (percentage of named alters targeted with advocacy related to any of the five areas of prevention asked about in the past 3 months; mean condom use advocacy across all alters; mean HIV testing advocacy across non-HIV-infected alters; and mean ART advocacy across all alters living with HIV). We did not include the PrEP and HIV care advocacy measures in our analysis due to lack of variance in responses and associated insufficient statistical power.

*Internalized HIV stigma* was measured using the 8-item Internalized AIDS-Related Stigma Scale [20]. Examples of items include “Being HIV-positive makes me feel like something is wrong with me” and “I feel guilty that I am HIV-positive.” Response options range from 1 “disagree strongly” to 5 “agree strongly.” The mean item score was calculated and higher scores represent greater stigma.

*HIV disclosure* was measured by calculating the *percentage of named alters whom the respondent reported having disclosed their HIV status to*. Also, respondents were asked to what extent they had shared their HIV status with sexual partners, family, and friends, in separate questions. Response options were 0 “none,” 1 “some,” and 2 “all,” and the mean item score was calculated to represent a *general measure of HIV disclosure*.

*HIV knowledge* was assessed with 13 statements related to the goals of HIV medication (e.g., “If a person with HIV infection does not take medication for HIV, their HIV viral load will increase”), drug resistance (e.g., “If you do not take HIV medication exactly as instructed, HIV in your body may become resistant to HIV medications”), adherence (e.g., “An HIV-positive individual does not need to take medication for HIV everyday if they do not have any symptoms”), HIV myths and misconceptions (e.g., “A person can get HIV through witchcraft of other supernatural means”), and HIV prevention (e.g., “Having an undetectable HIV viral load makes it very difficult for you to transmit the virus to someone else”). Some items were developed by the study team, while others were derived from the Patient’s HIV Knowledge Questionnaire [21]. Response options consisted of “true,” “false,” “don’t know,” and “not sure,” and a sum of correct responses was calculated.

*Positive living* was assessed with measures of condom use and ART adherence. Participant *condom use* in the past 6 months was assessed by asking participants to report how frequently they used condoms with their partner during sexual intercourse, using responses ranging from 1 “never” to 5 “always.” Condom use with a main partner was assessed, as well as with all sexual partners (if casual partners were present), in separate items. A binary variable was created to represent whether the participant reported consistent condom use (reported always using condoms) with all sex partners (main and/or casual) in the past 6 months. We assessed *ART adherence* using a single item in which participants rated the percentage of prescribed ART doses taken in the past month, on a scale of 0–100%. A binary variable was created to represent good adherence by indicating whether adherence was estimated to be 100%.

*Self-efficacy for HIV prevention advocacy* was measured with a single item in which participants rated their confidence in being able to start a conversation about HIV with people they know on a scale of 0 “cannot do at all” to 10 “certain I can do.”

*Socio-demographic characteristics* included age, gender, education, and relationship status. For analysis, binary derived variables were created to represent whether the participant was male, had any history of secondary education, and reported being in a committed relationship. *HIV medical characteristics* included time since HIV diagnosis, time in HIV care, and most recent CD4 cell count and HIV viral load, all of which were abstracted from the participant’s medical chart.

### Data Analysis

Spearman rho correlation coefficients were used to examine correlations between the measures of HIV prevention advocacy and measures of the constructs in the conceptual framework, as well as correlations among the constructs in the conceptual framework. We chose Spearman rho coefficients over Pearson coefficients because several variables were not normally distributed. Multiple linear regression analysis was then conducted to further examine correlates of HIV prevention advocacy, with separate models conducted for the general measure of HIV prevention advocacy and the percent of named alters who were targeted with any HIV prevention advocacy. In these models, independent variables consisted of internalized HIV stigma, percentage of named alters who the index participant disclosed to, HIV knowledge, consistent condom use, and HIV prevention advocacy self-efficacy. IBM SPSS Statistics, version 28.0.1, was used to conduct the analysis.

## Results

### Sample Characteristics

A sample of 311 PLWH were screened for study eligibility, of whom 287 were eligible and 210 enrolled (i.e., provided written informed consent and completed the baseline survey). Common reasons why eligible candidates chose not to enroll included lack of time to attend study visits and not having social network members whom they felt comfortable recruiting and/or who would agree to participate. Table 1 shows the characteristics of the enrolled participants. Most participants were female (70.0%) and average age was 40.0 years (SD = 10.6; range 19–69). Mean time in HIV care was 11.0 years (SD = 5.6), all were on ART, and the most recent HIV viral load was undetectable for 91.4%. The mean number of social network members (i.e., alters) named in the social network assessment was 15.3 (SD = 4.8), and mean percent of named alters who were believed to be living with HIV was 19.4% (SD = 16.0). Among the named alters who were believed to be living with HIV, 90.2% were perceived to be both in HIV care and on ART.

**Table 1:** Sample characteristics at baseline (*n* = 210)

	**Mean (SD)/ *N* (%)**
**Demographics**	
Mean age	40.0 (10.6)
Any secondary education	125 (59.5%)
Male	63 (30.0%)
In a committed relationship	139 (66.2%)
**HIV disease characteristics**	
Mean years since HIV diagnosis	13.4 (5.6)
Mean years in HIV care	11.0 (5.6)
Mean CD4 count (cell/mm^3^)	534 (298)
Last HIV viral load was undetectable	192 (91.4%)
On HIV antiretroviral therapy	210 (100%)
**Conceptual framework constructs**	
Mean internalized HIV stigma (possible range: 1–5)	2.31 (0.94)
Mean HIV disclosure	
General HIV disclosure (possible range: 0–2)	1.09 (0.37)
% of alters that participant disclosed their HIV status to	51.3 (32.3)
Mean HIV knowledge (possible range: 0–13)	10.58 (1.34)
Positive living	
Always use condoms with main and casual partners^a^	53 (25.0%)
Self-report 100% ART adherence in past month	136 (64.8%)
Mean HIV prevention advocacy self-efficacy (possible range: 0–10)	6.97 (2.15)
**HIV prevention advocacy**	
Mean general HIV prevention advocacy (possible range: 1–5)	2.33 (0.88)
Mean percent of alters targeted with HIV prevention advocacy	30.6 (26.9)
Mean condom use advocacy across all alters (possible range: 0–1)	0.09 (0.13)
Mean HIV testing advocacy across all non-HIV+ alters (possible range: 0–1)	0.11 (0.16)
Mean PrEP advocacy across all non-HIV+ alters (possible range: 0–1)	0.01 (0.07)
Mean HIV care advocacy across all HIV+ alters (possible range: 0–1)	0.21 (0.28)
Mean ART adherence advocacy across all HIV+ alters (possible range: 0–1)	0.26 (0.28)

*SD* standard deviation

^a^*N* = 132 with a main partner and/or casual partners in past 6 months

### Prevalence of HIV Prevention Advocacy and Correlates of Advocacy Among Constructs in the Conceptual Framework

The general measure of HIV prevention advocacy revealed a moderate level of advocacy [mean (SD) = 2.33 (0.88)] during the past 3 months. About a third (30.6%) of the named alters had been targeted in the prior 3 months with discussions about any of the five HIV prevention areas of advocacy that we enquired about. The most common targets of advocacy were HIV care engagement (35.3%) and ART adherence (44.0%) among alters living with HIV; 15.5% of all alters were targeted with condom use advocacy, and 18.0% and 2.5% of alters not living with HIV were targeted with HIV testing and PrEP advocacy, respectively. The mean level of advocacy across all relevant alters related to each of the five topical areas for prevention was low (means range from 0.01 to 0.25; potential range of 0–2) (see Table 1).

The data revealed a wide range of values for each of the constructs in our conceptual framework for understanding factors contributing to engagement in HIV prevention advocacy. Moderate levels of internalized HIV stigma, HIV disclosure, positive living (consistent condom use, ART adherence), and HIV prevention self-efficacy were found, while HIV knowledge was high (see Table 1). Table 2 lists how each of the constructs in the framework correlates with each measure of HIV prevention advocacy. *Internalized HIV stigma* was negatively correlated with the general measure of HIV prevention advocacy. *Percent of named alters to whom the participant had disclosed their HIV status* was positively correlated with the percent of alters targeted with any HIV prevention advocacy and the mean level of ART adherence-related advocacy among alters living with HIV. The *general measure of HIV disclosure* was not correlated with any of the advocacy measures. *HIV knowledge* was positively correlated with percent of alters targeted with any prevention advocacy, and the mean levels of HIV testing and condom use advocacy among relevant alters. Among measures of *positive living,* consistent condom use had positive correlations with each measure of HIV prevention advocacy, except ART adherence-related advocacy, while good ART adherence was not associated with any of the advocacy measures. Lastly, *HIV prevention advocacy self-efficacy* was positively correlated with the general measure of HIV prevention advocacy, percent of alters targeted with any advocacy, and mean level of HIV testing advocacy across alters not living with HIV.

**Table 2:** Correlates (Spearman rho coefficient; *p* value) of multiple measures of HIV prevention advocacy

	General HIV prevention advocacy	Percent of alters targeted with HIV-related advocacy	Mean HIV testing advocacy across all HIV- alters	Mean condom use advocacy across all alters	Mean ART advocacy across all HIV+ alters
Internalized HIV stigma	**− .15 (.04)**	− .02 (.74)	− .02 (.75)	.04 (.57)	.02 (.81)
General HIV disclosure	.08 (.28)	.06 (.37)	− .02 (.77)	− .04 (.62)	.04 (.60)
Percent of alters disclosed to	.10 (.17)	**.16 (.02)**	.03 (.69)	− .02 (.73)	**.15 (.04)**
HIV knowledge	.12 (.09)	**.20 (.003)**	**.23 (< .001)**	**.26 (< .001)**	.08 (.29)
Consistent condom use	**.27 (< .001)**	**.20 (.01)**	**.23 (.004)**	**.23 (.005)**	.07 (.44)
Good ART adherence	.05 (.47)	− .01 (.92)	.09 (.22)	− .05 (.49)	.03 (.71)
HIV prevention advocacy self-efficacy	**.25 (< .001)**	**.18 (.009)**	**.14 (.04)**	.10 (.13)	-.08 (.31)

Correlation coefficients that are statistically significant (*p* < .05) are bolded

We used multiple linear regression analysis to further examine correlates of general HIV prevention advocacy and percent of alters who were targeted with any advocacy (see Table 3). In the model for general HIV prevention advocacy, independent correlates consisted of HIV knowledge [std. beta (SE) = 0.15 (0.05); *p* = .04], consistent condom use [std. beta (SE) = 0.26 (0.15); *p* < .001], and self-efficacy for HIV prevention advocacy [std. beta (SE) = 0.18 (0.03); *p* = .02], after controlling for all dependent variables. In the model for percent of alters who were targeted with any advocacy, independent correlates consisted of percent of alters to whom the index participant had disclosed their HIV status [std. beta (SE) = 0.19 (0.07); *p* = .02], HIV knowledge [std. beta (SE) = 0.21 (1.60); *p* = .01], and consistent condom use [std. beta (SE) = 0.16 (4.92); *p* = .04].

**Table 3:** Multiple linear regression models of correlates of measures of HIV prevention advocacy

	Percent of alters targeted with any HIV prevention advocacy	General HIV prevention advocacy
	Std. beta (SE); *p*	Std. beta (SE);* p*
Internalized HIV stigma	0.02 (2.46); .82	− 0.15 (0.07); .07
Percent of alters disclosed to	**0.19 (0.07); .02**	− 0.03 (0.002); .70
HIV knowledge	**0.21 (1.60); .01**	**0.15 (0.05); .04**
Consistent condom use	**0.16 (4.92); .04**	**0.26 (0.15); < .001**
HIV prevention advocacy self-efficacy	0.12 (1.00); .15	**0.18 (0.03); .02**

Standardized beta coefficients that are statistically significant (*p* < .05) are bolded

*Std* standardized, *SE* standard error

### Correlates Among Measures in the Conceptual Framework

Table 4 lists the correlations among the constructs we hypothesized to be associated with advocacy. *Internalized HIV stigma* was negatively correlated with both the general measure of HIV disclosure and percent of alters disclosed to, as well as HIV prevention self-efficacy. Aside from stigma, the two measures of *HIV disclosure* were positively correlated with each other. However, neither was significantly associated with other constructs. *HIV knowledge* was positively correlated with consistent condom use. The two measures of *positive living*, consistent condom use and good ART adherence, were correlated with each other, and consistent condom use was positively correlated with HIV knowledge, as noted above. *HIV prevention advocacy self-efficacy* was correlated with internalized HIV stigma as noted above.

**Table 4:** Correlations (Spearman rho coefficient; *p* value) among constructs that are hypothesized to influence HIV prevention advocacy

	Internalized HIV stigma	General HIV disclosure	Percent of alters disclosed to	HIV knowledge	Consistent condom use	Good ART adherence
Internalized HIV stigma	--	--	--	--	--	--
General HIV disclosure	**− .37 (< .001)**	--	--	--	--	--
Percent of alters disclosed to	**− .25 (< .001)**	**.34 (< .001)**	--	--	--	--
HIV knowledge	.05 (.47)	− .02 (.74)	− .12 (.09)	--	--	--
Consistent condom use	− .05 (.53)	.06 (.43)	.08 (.35)	**.17 (.04)**	--	--
Good ART adherence	− .06 (.36)	− .04 (.61)	.00 (.99)	.10 (.15)	**.18 (.03)**	--
HIV prevention advocacy self-efficacy	− **.28 (< .001)**	.12 (.08)	.03 (.71)	.05 (.47)	.16 (.051)	.02 (.74)

Correlation coefficients that are statistically significant (*p* < .05) are bolded

## Discussion

In this sample of PLWH who are receiving HIV care, each of the constructs in our conceptual model of engagement in HIV prevention advocacy was correlated with advocacy, as hypothesized. At least one of the general or alter-specific measures of HIV prevention advocacy was correlated with internalized HIV stigma, HIV disclosure, HIV knowledge, positive living, and HIV prevention advocacy self-efficacy. Multiple regression analysis revealed that HIV knowledge, consistent condom use (a measure of positive living), and self-efficacy for advocacy may be particularly important for engagement in HIV prevention advocacy.

Our data revealed low-to-moderate levels of engagement in HIV prevention advocacy. The general measure of advocacy showed moderate levels of advocacy, and respondents reported discussing HIV with about a third of their named social network members. However, engagement in specific target areas of advocacy (i.e., HIV testing, condom use, HIV care, ART adherence, PrEP use) was low among named alters. Prior studies of HIV prevention advocacy among PLWH in Uganda have shown that nearly everyone engages in advocacy on some level [6, 7, 17]. However, this study is among the first to describe the frequency and breadth of advocacy within one’s social network, by examining the proportion of social network members targeted with advocacy and the HIV prevention content areas of advocacy.

Our conceptual model posits that low internalized HIV stigma is important for being comfortable with HIV disclosure and sharing one’s experience of living with HIV, which we consider critical for establishing a foundation of engagement in HIV prevention advocacy. As we hypothesized, internalized HIV stigma was negatively correlated with HIV disclosure, which is consistent with other research [13]. The level of HIV disclosure among named alters was associated with HIV prevention advocacy in our data, including being an independent correlate of the level of advocacy within the participants’ social networks after controlling for other correlates. Other studies have also found a significant association between HIV disclosure and HIV prevention advocacy [14]. These findings highlight the need for advocacy training interventions to first focus on reducing internalized stigma and increasing comfort with HIV disclosure and disclosure decision-making. This sets the foundation for being empowered to engage in HIV prevention advocacy.

Greater HIV knowledge was positively correlated with most measures of HIV prevention advocacy, and multiple regression analysis revealed it to be an independent correlate of both the general and alter-specific measures of advocacy. Being knowledgeable about various aspects of HIV may enable someone to feel more comfortable and confident to engage in advocacy, knowing that they have the knowledge to provide accurate information during advocacy. This finding about the importance of knowledge for engagement in health behavior advocacy may apply to other health contexts. Of note, these findings are consistent with our research in the context of peer advocacy for cervical cancer screening in Uganda, which also found that greater knowledge was a strong correlate of level of engagement in advocacy [22].

Positive living—specifically consistent condom use—was positively associated with most measures of HIV prevention advocacy and was independently correlated with both the general and alter-specific measures of advocacy after controlling for other correlates. Prior research has also found that advocates who report consistent condom use are apt to engage in more HIV prevention advocacy, including condom use advocacy [4, 5]. Furthermore, consistent condom use was positively correlated with greater self-efficacy for advocacy. These findings are consistent with our hypothesis that people are more apt to feel comfortable engaging in advocacy for a behavior that they themselves engage in.

Consistent with self-efficacy being an important precursor to behavioral activation [16], our data showed that self-efficacy or confidence in initiating discussions about HIV (i.e., advocacy) was positively correlated with both the general and alter-specific measures of HIV prevention advocacy in the adjusted multiple regression analysis. Self-efficacy for HIV prevention advocacy was negatively correlated with internalized HIV stigma (as we had hypothesized), as well as positively correlated with consistent condom use. These findings provide further support for how the constructs within the model are inter-related in their influence on engagement in advocacy.

There are limitations to our analysis. The PLWH in our sample reflect selection bias, as they had decided to enroll in a study that would train them to engage in HIV prevention advocacy. Motivation to be such an advocate is likely associated with lower internalized stigma and greater levels of HIV knowledge and HIV disclosure, and not representative of PLWH in general. Also, this analysis was cross-sectional, which precludes any causal inferences.

In conclusion, the findings from this cross-sectional analysis provide support for the validity of our conceptual model regarding constructs associated with engagement in HIV prevention advocacy among PLWH. These findings also provide support for the design and composition of the intervention we are currently testing with this sample. The study’s post-intervention follow-up data will enable us to further explore the relationships examined in this analysis. Future analyses will evaluate the intervention effects on each construct hypothesized in our conceptual framework to drive engagement in HIV prevention advocacy, and the effects of increased advocacy on HIV protective behaviors among social network members.

## Data Availability

De-identified dataset and statistical code are available to researchers upon submission of proposal and review by the study team.
